# Hand to Mouth: A Systematic Review and Meta-Analysis of the Association between Rheumatoid Arthritis and Periodontitis

**DOI:** 10.3389/fimmu.2016.00080

**Published:** 2016-03-02

**Authors:** Nicholas R. Fuggle, Toby O. Smith, Arvind Kaul, Nidhi Sofat

**Affiliations:** ^1^Musculoskeletal Research Group, Institute of Infection and Immunity, St George’s University of London, London, UK; ^2^Faculty of Medicine and Health Sciences, University of East Anglia, Norwich, UK; ^3^Department of Rheumatology, St George’s University Hospitals NHS Foundation Trust, London, UK

**Keywords:** rheumatoid arthritis, inflammatory arthritis, periodontitis, periodontal disease, meta-analysis

## Abstract

**Background:**

Rheumatoid arthritis (RA) and periodontitis are both chronic inflammatory diseases, which demonstrate similarities in terms of mechanism, histopathology, and demography. An association between these conditions has been demonstrated previously but has been called into question more recently.

**Methods:**

The published databases, such as MEDLINE, EMBASE, and PsycINFO, were searched using search terms related to RA and periodontitis. Articles were selected if they included data on the number of people with RA diagnosed with periodontitis (or periodontal disease parameters) compared to a control comparison group. Review articles, case reports, animal model studies, non-English language, and articles with unavailable abstracts were excluded. Data were extracted, critically appraised using the Downs and Black tool, and a random-effect Mantel–Haenszel meta-analysis was performed.

**Results:**

Twenty-one papers met the eligibility criteria and provided data for the meta-analysis; 17 studies (including a total of 153,492 participants) comparing RA to healthy controls and 4 (including a total of 1378 participants) comparing RA to osteoarthritis (OA). There was a significantly increased risk of periodontitis in people with RA compared to healthy controls (relative risk: 1.13; 95% CI: 1.04, 1.23; *p* = 0.006; *N* = 153,277) with a significantly raised mean probing depth, risk of bleeding on probing (BOP), and absolute value of clinical attachment loss in those with RA. When comparing RA and OA, there was no significant difference in the prevalence of periodontitis; however, the risk of BOP was greater in OA than RA.

**Conclusion:**

A significant association between RA and periodontitis is supported by the results of our systematic review and meta-analysis of studies comparing RA to healthy controls. In our meta-analysis, however, this is not replicated when comparing RA to OA controls.

## Introduction

Rheumatoid arthritis (RA) is an autoimmune disease characterized by joint inflammation and destruction leading to chronic disability, early mortality, systemic complications, and high socioeconomic burden on society as a whole (1). It has a prevalence of 0.5–1.0% in US populations (2). The exact etiology of RA is unknown; however, it is thought to be secondary to an interaction between genetic attributes and environmental exposures, as demonstrated by the established association with smoking (3) and genetic polymorphisms, including HLA-DRB1 (4).

Auto-antibodies to the Fc portion of immunoglobulin are known as rheumatoid factor, and antibodies that form against citrullinated proteins are called anti-citrullinated protein antibody (ACPA) or anti-cyclic citrullinated peptide (anti-CCP). If either of these are present then they confer “seropositivity” that is seen in 70–80% of patients (5) and are associated with more aggressive disease and with the earlier development of erosions. Rheumatoid factor is limited in diagnostic application by low specificity; however, ACPA is 95–98% specific (6).

Anti-citrullinated protein antibody has been shown to be ­present in RA patient sera up to a decade prior to the development of the disease (7), although the amount of ACPA and inflammatory cytokine levels rises sharply a few months before the synovitis presents (8). It is therefore hypothesized that as well as citrullination of endogenous proteins, a second inflammatory “hit” is required to stimulate the development of RA. Citrullinated proteins are also associated with other environmental factors such as smoking and pathological conditions, including periodontitis.

Peptidyl arginine deiminase (PAD) causes the post-­translational modification of arginine to citrulline. It is hypothesized that this citrullination leads to amino acid chains being recognized as auto-antigens, which leads to the development of auto-antibodies and the subsequent autoimmune damage that is the signature for RA. PAD is produced by human cells, for example, in the lung; however, it is also produced by the microbe *Porphyromonas gingivalis* (9).

Peptidyl arginine deiminase production by *P. gingivalis*, an anaerobic prokaryote, has been demonstrated *in vitro*. Due to this organism’s role in the development of periodontal disease and the association of RA with periodontitis, it has been hypothesized that *P. gingivalis* provides a causal link among periodontal disease, citrullination, and RA (10).

Periodontitis is a destructive, infectious-inflammatory condition affecting the gums. It is characterized by loss of gingival attachment between the tooth and the gingivae leading to the formation of a periodontal pocket (11). Initially, a biofilm structure develops, which causes localized inflammation in the form of gingivitis. This biofilm is then colonized by anaerobic bacteria that cause further inflammation and neutrophillic activation. Matrix metalloproteinases are spilled during this inflammatory reaction leading to tissue destruction and further exacerbate the attachment loss and deepening the periodontal pocket, resulting in further anaerobic colonization, soft tissue destruction, alveolar bone loss, and, ultimately, tooth loss.

The prevalence of periodontitis varies internationally; however, approximately 10–15% of the global adult population are affected by the condition (12). Known risk factors include smoking, age, diabetes mellitus (13), educational level (14), and immunological diseases (e.g., HIV) (15). Periodontitis, itself is associated with a higher risk of stroke, cardiovascular disease (16), and pneumonia (17).

It is divided into two subtypes; aggressive periodontitis and chronic periodontitis (CP). CP is the more common form affecting an older population with an indolent disease progression; however, aggressive periodontitis is observed in a young population, aggregates in families, and leads to rapid tooth loss and the need for prostheses (18).

Certain bacteria have been implicated in the development of periodontitis, including the “red complex” organisms; *P. gingivalis*, *Tannerella forsythia*, *Treponema denticola*, and *Aggregatibacter actinomycetemcomitans*. It is not clear whether there are microbiological differences between the chronic and aggressive subtypes of periodontitis (18).

Epidemiological studies have shown a strong association between periodontitis and RA (19, 20), though these have been hampered by their cross-sectional nature, variability in definition of periodontitis and dental endpoints, the extent of oral examination, and the limited RA information collected.

Shared risk factors, including cigarette smoke, provide possible confounders; however, an increased risk of periodontitis has been demonstrated in a non-smoking RA group (21). In addition, it appears that periodontitis responds to RA treatment (22). There are also pathological similarities between the two conditions in terms of T cell activation, inflammatory cytokine profile and the resultant bone destruction and deformity.

The association between the two diseases has recently been debated with the relevance of *P. gingivalis* being called into question in a large cohort of pre-RA participants (23). Recently, there have been narrative reviews of the literature surrounding the association between periodontitis and RA (24–26). However, there is no recent systematic review or meta-analysis to re-evaluate this association. Our aim was to analyze the association between these two conditions in light of all recent evidence in the form of a meta-analysis and to include all the most recent published studies investigating the association of RA and periodontal disease worldwide to assess the largest dataset possible for meta-analysis.

## Materials and Methods

### Search Strategy

We searched the published databases: MEDLINE *via* OVID, EMBASE *via* OVID, and PsycINFO *via* OVID. No restrictions were placed on date of publication; however, only English language articles were selected. Articles were searched for using the terms “periodontitis,” “periodontitis.mp,” or “periodontal disease” and “rheumatoid arthritis.mp” or “inflammatory arthritis.” All papers which presented data on the number of people with RA diagnosed with periodontitis or periodontal disease measures/assessments, compared to a comparison group [such as healthy controls or those with osteoarthritis (OA)] were included. Review articles, case reports, animal model studies, and those with unavailable abstracts were excluded from the analysis. The titles and abstracts of each citation were independently reviewed by two authors (Nicholas Rubek Fuggle and Toby O. Smith) and verified by a third (Nidhi Sofat). Full texts of all potentially eligible papers were independently reviewed by three authors (Nicholas Rubek Fuggle, Toby O. Smith, and Nidhi Sofat) with consensus made through discussion on final study eligibility.

### Data Extraction

Data were extracted onto a predefined data extraction table. Data extracted included participant number with RA and a comparator (e.g., non-RA healthy controls or OA), age, gender, ethnic origin, marital status, educational status, smoking history, medical history/status (cardiovascular disease, diabetes obesity, osteoporosis, antibiotic usage), RA diagnosis, years of disease (if RA), measures of disease severity (e.g., rheumatoid factor, CRP, ESR, HLA-DRB1), current therapies (e.g., DMARDs, NSAIDS, biologics), and periodontitis measure. These included percentage with periodontitis, probing depth, plaque index, missing teeth, proportion of sites with plaque, bleeding on probing (BOP), and clinical attachment loss (CAL). All data were extracted by two reviewers independently (Nicholas Rubek Fuggle and Toby O. Smith) with any disagreements addressed through discussion.

### Critical Appraisal

All included studies were assessed using the Downs and Black critical appraisal tool for non-randomized controlled trials (27). This is a 27-item critical appraisal tool that assessed study quality (10 items), external validity (3 items), study bias (7 items), confounding and selection bias (6 items), and power of the study (1 item). All papers were independently assessed by one reviewer (Nicholas Rubek Fuggle) and verified by a second reviewer (Toby O. Smith).

### Data Analysis

Study heterogeneity was assessed through visual assessment of the data extraction table. As there was homogeneity in participant characteristics, periodontal assessment, and study design, a meta-analysis was undertaken. The primary analysis was to estimate the relative risk (RR) of periodontitis for people with RA over people without RA, or another non-RA condition such as OA. The secondary analyses included estimating the RR or mean difference (MD) for measures of periodontal disease between patients with RA and comparative groups (non-RA or OA). Periodontal disease measures included probing depth, plaque index, missing teeth, proportion of sites with plaque, BOP, and CAL. A fixed-effect Mantel–Haenszel meta-analysis was undertaken when the inconsistency value (*I*^2^) was 50% or less and Chi^2^ equated *p* ≥ 0.10. A random-effect Mantel–Haenszel meta-analysis was undertaken when *I*^2^ was >50% and Chi^2^ equated to *p* < 0.10. All analyses were calculated with 95% confidence intervals and forest-plots were constructed, and performed on RevMan Version 5.3 (Copenhagen: The Nordic Cochrane Centre, The Cochrane Collaboration, 2014).

## Results

### Search Results

**Figure 1:**
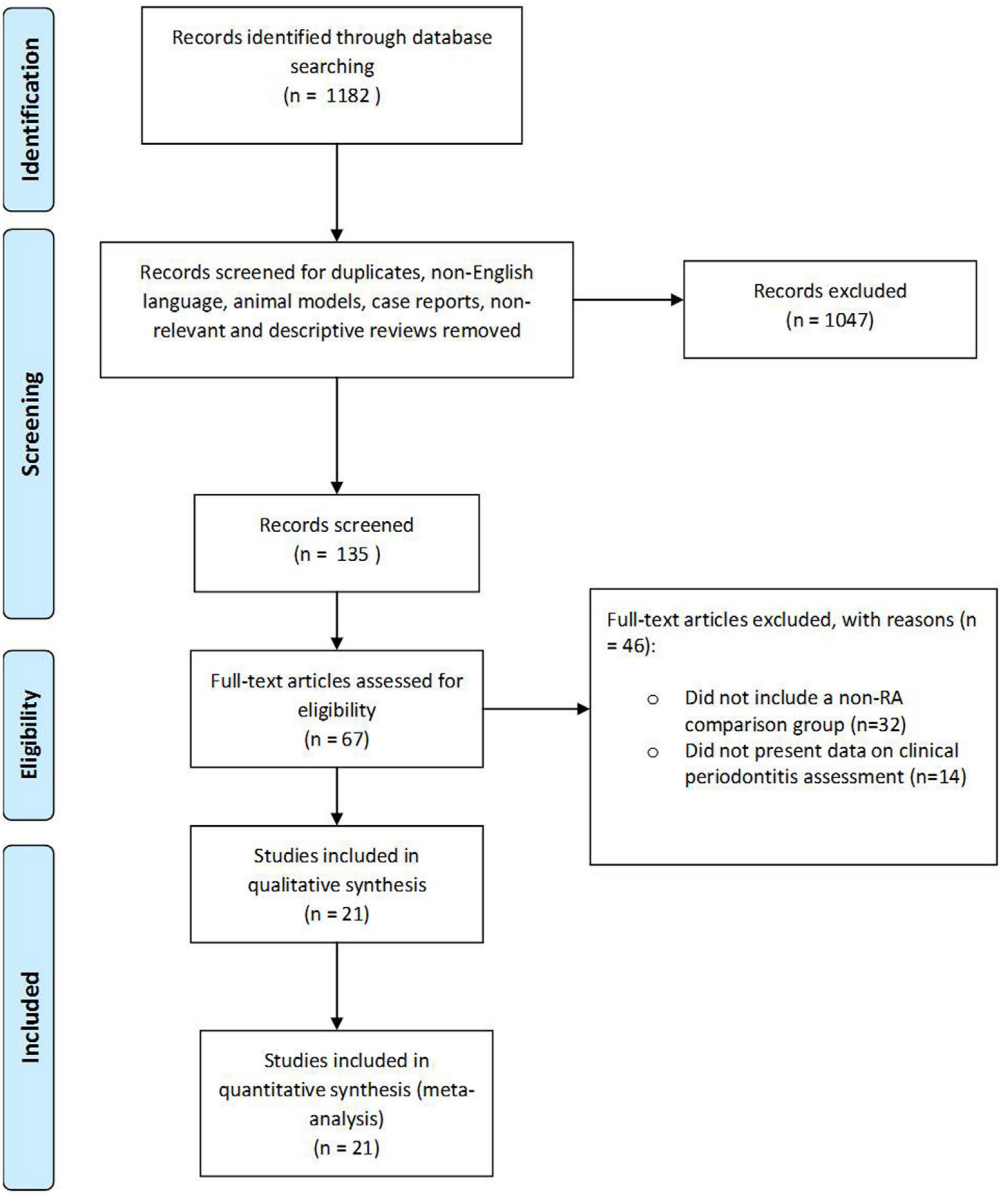
**PRISMA flow-chart depicting the results of the search strategy**.

A summary of the search results is presented in Figure 1. As this illustrates, from a total of 1182 citations identified from the search strategy, 67 provisionally met the inclusion criteria. In total, 21 papers met the eligibility criteria and provided data for the meta-analysis.

### Characteristics of Included Studies

The characteristics of the participants from the included studies are presented in Table 1. Seventeen studies analyzed data on periodontitis in RA compared to non-RA healthy control cohorts (21, 28–43). Four studies analyzed data on periodontitis in RA compared to OA cohorts (10, 44–46).

**Table 1:** **Characteristics of included studies**.

Reference	*N* (RA/comparison)	Comparison group	Mean age (RA/comparison)	Gender (% female)	Current smokers (RA/comparison %)	Ex-smokers (RA/comparison %)	Years of RA disease	RA rheumatoid factor **+** (%)	DAS28
Abou-Raya et al. (36)	50/50	Non-RA control	52.5/52	80/80	N/S	N/S	13.5	86	4.9
Bıyıkoğlu et al. (38)	30/15	Non-RA control	52.7/46.6	N/S	N/S	N/S	N/S	N/S	N/S
Bıyıkoğlu et al. (13)	23/17	Non-RA control	52.7/40.7	78/47	22/35	N/S	16.3	26	N/S
Chen et al. (30)	13,779/137,790	Non-RA control	52.6/52.4	77/77	N/S	N/S	N/S	N/S	N/S
Coburn et al. (44)	287/330	OA	57/58	72/78	15/8	31/23	N/S	N/S	N/S
de Smit et al. (33)	95/44	Non-RA control	56/34	68/57	23/27	40/43	7.4	53	2.4
Dissick et al. (46)	69/35	OA	62/58	17/14	20/17	45/29	14	56	N/S
Esen et al. (41)	20/20	Non-RA control	40.1/44.4	80/95	N/S	10/5	N/S	N/S	3.2
Janssen et al. (31)	86/36	Non-RA control	57/26	56/59	60/42	17/36	N/S	74	2.2
Joseph et al. (39)	100/112	Non-RA control	46.5/45.9	76/86	N/S	N/S	N/S	N/S	N/S
Lee et al. (35)	248/85	Non-RA control	60.1/59.1	88/87	2/5	4/5	N/S	N/S	N/S
Reichert et al. (28)	42/114	Non-RA control	56.1/53.8	22/26	6/3	5/4	N/S	N/S	N/S
Mercado et al. (42)	65/65	Non-RA control	56.4 (total)	75 (total)	7/7	N/S	N/S	N/S	N/S
Mikuls et al. (10)	287/330	OA	59/59	37/40	19/11	43/35	12.6	77	3.2
Mirrielees et al. (32)	35/35	Non-RA control	46.8/43	77/74	11/0	N/S	N/S	N/S	N/S
Pischon et al. (40)	57/52	Non-RA control	52.1/52.1	86/83	21/19	39/21	N/S	N/S	N/S
Potikuri et al. (21)	91/93	Non-RA control	43.9/41.8	84/74	N/S	N/S	2.0	63	7.2
Scher et al. (37)	31/18	Non-RA control	42.2/42.2	68/65	16/6	16/16	3.4 months	92	5.8
Susanto et al. (43)	75/75	Non-RA control	46.5/46.9	80/80	7/7	N/S	N/S	N/S	N/S
Témoin et al. (45)	11/25	OA	62.6/75.0	N/S	N/S	N/S	N/S	N/S	N/S
Wolff et al. (29)	22/22	Non-RA control	51.7/51.9	68/68	3/3	10/10	5.9 months	37	4.6

*DAS28, disease activity score 28; N/S, not stated; OA, osteoarthritis; RA, rheumatoid arthritis*.

In the RA compared to non-RA analysis, a total of 153,492 participants were analyzed. This consisted of 14,849 people with RA, compared to 138,643 non-RA control participants. Mean age of the RA cohort was 50.9 (SD: 5.7) years compared to 46.4 years (SD: 8.3). In the RA group, duration of RA disease was stated in eight studies (10, 21, 29, 33, 34, 36, 37, 46), ranging from 3.4 months (37) to 16.3 years (34).

In the RA compared to OA analysis, 1378 participants were analyzed. This consisted of 654 people with RA were compared to 724 individuals with OA. Mean age of the RA cohort was 60.2 (SD: 2.6) years compared to 62.5 years (SD: 8.3).

### Critical Appraisal

The results of the critical appraisal assessment are presented in Table 2.

**Table 2:** **Downs and Black critical appraisal results**.

Reference	Downs and Black appraisal criteria																										
	1	2	3	4	5	6	7	8	9	10	11	12	13	14	15	16	17	18	19	20	21	22	23	24	25	26	27
Abou-Raya et al. (36)	✓	✓	✓	✓	X	✓	X	N	N	✓	X	X	X	N	✓	N	X	✓	N	✓	✓	✓	N	N	X	N	✓
Bıyıkoğlu et al. (38)	✓	✓	✓	✓	✓	✓	X	N	N	✓	✓	✓	✓	N	X	N	X	✓	N	✓	X	X	N	N	X	N	X
Bıyıkoğlu et al. (34)	✓	✓	✓	✓	✓	✓	X	N	N	✓	✓	✓	✓	N	X	N	X	✓	N	✓	X	X	N	N	X	N	X
Chen et al. (30)	✓	✓	✓	✓	✓	✓	✓	N	N	✓	✓	✓	✓	N	X	N	X	✓	N	✓	X	X	N	N	✓	N	✓
Coburn et al. (44)	✓	✓	✓	✓	✓	✓	X	N	N	✓	✓	✓	✓	N	✓	N	X	✓	N	✓	✓	✓	N	N	✓	N	✓
de Smit et al. (33)	✓	✓	✓	✓	✓	✓	✓	N	N	✓	✓	✓	✓	N	X	N	X	✓	N	✓	X	X	N	N	X	✓	X
Dissick et al. (46)	✓	✓	✓	✓	✓	✓	✓	N	N	✓	✓	✓	✓	N	✓	N	X	✓	N	✓	✓	X	N	N	X	N	X
Esen et al. (41)	✓	✓	✓	✓	✓	✓	✓	N	N	✓	✓	✓	✓	N	X	N	X	✓	N	✓	✓	✓	N	N	X	N	X
Janssen et al. (31)	✓	✓	✓	✓	✓	✓	X	N	N	✓	✓	X	X	N	X	N	X	✓	N	✓	X	X	N	N	X	N	X
Joseph et al. (39)	✓	✓	✓	✓	✓	✓	X	N	N	✓	✓	X	✓	N	X	N	X	✓	N	✓	X	X	N	N	✓	N	✓
Lee et al. (35)	✓	✓	✓	✓	✓	✓	X	N	N	✓	X	X	X	N	X	N	X	✓	N	✓	X	X	N	N	✓	N	✓
Mercado et al. (42)	✓	✓	✓	✓	✓	✓	✓	N	N	✓	✓	✓	✓	N	X	N	X	✓	N	✓	✓	X	N	N	X	N	X
Mikuls et al. (10)	✓	✓	✓	✓	✓	✓	✓	N	N	✓	✓	✓	✓	N	✓	N	X	✓	N	✓	✓	✓	N	N	✓	N	✓
Mirrielees et al. (32)	✓	✓	✓	✓	✓	✓	X	N	N	✓	✓	✓	✓	N	X	N	X	✓	N	✓	✓	✓	N	N	X	N	X
Reichert et al. (28)	✓	✓	✓	✓	X	✓	X	N	N	✓	X	X	✓	N	X	N	X	✓	N	✓	X	X	N	N	X	N	✓
Pischon et al. (40)	✓	✓	✓	✓	✓	✓	✓	N	N	✓	✓	✓	✓	N	X	N	X	✓	N	✓	X	X	N	N	X	N	✓
Potikuri et al. (21)	✓	✓	✓	✓	✓	✓	X	N	N	✓	X	X	X	N	X	N	X	✓	N	✓	X	X	N	N	✓	N	✓
Scher et al. (37)	✓	✓	✓	✓	X	✓	X	N	N	✓	✓	✓	✓	N	X	N	X	✓	N	✓	X	X	N	N	X	N	X
Susanto et al. (43)	✓	✓	✓	✓	✓	✓	✓	N	N	✓	✓	✓	✓	N	X	N	X	✓	N	✓	✓	X	N	N	X	N	✓
Témoin et al. (45)	✓	✓	✓	✓	X	✓	X	N	N	✓	X	X	X	N	X	N	X	✓	N	✓	X	X	N	N	X	N	X
Wolff et al. (29)	✓	✓	✓	✓	✓	✓	✓	N	N	✓	✓	✓	✓	N	X	N	X	✓	N	✓	X	X	N	N	X	N	X

*✓, satisfied; X, not satisfied; N, not applicable*.

*1. Hypotheses/aims/objectives clearly stated*.

*2. Main outcome measures clearly described*.

*3. Characteristics of patients/subjects clearly described*.

*4. Interventions of interest clearly described*.

*5. Distribution of principal confounders in each group clearly described*.

*6. Main findings clearly described*.

*7. Estimates of random variability in the data provided*.

*8. Important adverse events reported*.

*9. Characteristics of patients lost to follow-up described*.

*10. Actual probability values reported*.

*11. Participants approached representative of entire population*.

*12. Participants recruited representative of entire population*.

*13. Staff, places, and facilities representative of majority of population*.

*14. Blinding of study subjects*.

*15. Blinding of assessors*.

*16. Data based on data-dredging clearly stated*.

*17. Adjustment of different length of follow-up or duration between case and control*.

*18. Appropriate statistical tests used*.

*19. Compliance to intervention reliable*.

*20. Main outcome measure reliable and valid*.

*21. Intervention groups or case–controls recruited from same population*.

*22. Intervention groups or case–controls recruited at the same time*.

*23. Study subjects randomized to the interventions*.

*24. Was concealed randomization to allocation undertaken*.

*25. Adequate adjustment made in the analysis of confounders*.

*26. Patient losses accounted for*.

*27. Sufficiently powered cohort size*.

The evidence-base presented for moderate evidence for both the analysis of RA versus non-RA cohorts and RA versus OA cohort periodontitis measures. Recurrent strengths to the evidence included presenting clear aims and objectives (*N* = 21; 100%), presenting outcome data (*N* = 21; 100%), and participant characteristics data clearly (*N* = 21; 100%), as well as clearly presenting information on periodontitis assessment performed (*N* = 21; 100%), using valid and reliable measures of assessment in all cases (*N* = 21; 100%). The included studies for both assessments also analyzed their data with the appropriate statistical tests (*N* = 21; 100%), provided actual probability values (*N* = 21; 100%), although only nine studies presented estimates on random variability from their data (10, 29, 30, 33, 40–43, 46).

However, recurrent weaknesses in the evidence included poorly blinding assessors to their pathological or non-pathological group, only masked in four studies (10, 36, 44, 46), recruiting cases and controls at the same point in time in only five studies (10, 32, 36, 41, 44), whereas only six studies adequately adjusted their analyses for important confounders such as smoking and alcohol history and antibiotic usage (10, 21, 30, 35, 39, 44). Only 10 papers recruited cohorts that consisted of 50 participants or more per group (10, 21, 30, 35, 36, 39, 40, 42–44). Finally, no studies adjusted their analysis based on different lengths of follow-up or duration between cases and control.

### Meta-Analysis: Risk Ratio of Periodontitis in RA versus Non-RA Participants

The results of the meta-analyses are presented in Table 3.

**Table 3:** **Results from the meta-analyses**.

Outcome	Relative risk (95% CI)	*p*-Value	*N*	Statistical heterogeneity (*I*^2^; Chi^2^)
**Ra versus non-RA**				
Periodontitis (frequency)	1.13 (1.04, 1.23)	0.006	153,277	95%; <0.001
Probing depth (frequency >5 mm)	4.93 (0.84, 28.95)	0.08	339	85%; 0.001
Probing depth (mm)	0.69 (0.26, 1.12)^a^	0.002	1072	98%; <0.001
Plaque index (frequency >0.6)	1.23 (0.81, 1.88)	0.33	379	65%; 0.04
Plaque index (mean)	2.27 (−0.16, 4.70)^a^	0.07	522	99%; <0.001
Any bleeding on probing (frequency)	2.65 (1.00, 7.02)	0.05	792	74%; <0.001
Clinical attachment loss (frequency)	3.63 (0.40, 32.88)	0.25	170	91%; <0.001
Clinical attachment loss (mm)	0.99 (0.38, 1.61)^a^	0.002	958	97%; <0.001
Gingivitis Index (mean)	0.30 (0.20, 0.41)^a^	<0.001	654	72%; 0.03
Loss teeth (mean)	2.46 (0.30, 4.63)^a^	0.03	495	80%; 0.002
Periodontal bone loss (frequency moderate-to-severe)	2.05 (1.40, 2.98)	<0.001	130	N/E
**Ra versus OA**				
Periodontitis (frequency)	1.10 (0.81, 1.49)	0.54	1344	70%; 0.02
Probing depth (frequency >5 mm)	1.11 (0.95, 1.31)	0.19	617	N/E
Any bleeding on probing (frequency)	0.92 (0.88, 0.97)	0.002	617	N/E
Clinical attachment loss (frequency)	1.04 (0.74, 1.48)	0.81	617	N/E
Loss teeth (mean)	−0.10 (−0.62, 0.42)^a^	0.71	617	N/E

*^a^Mean difference analysis as opposed to relative risk analysis*.

*CI, confidence intervals; *I*^2^, inconsistency value; *N*, number of participants; N/E, not estimatable; OA, osteoarthritis; RA, rheumatoid arthritis*.

**Figure 2:**
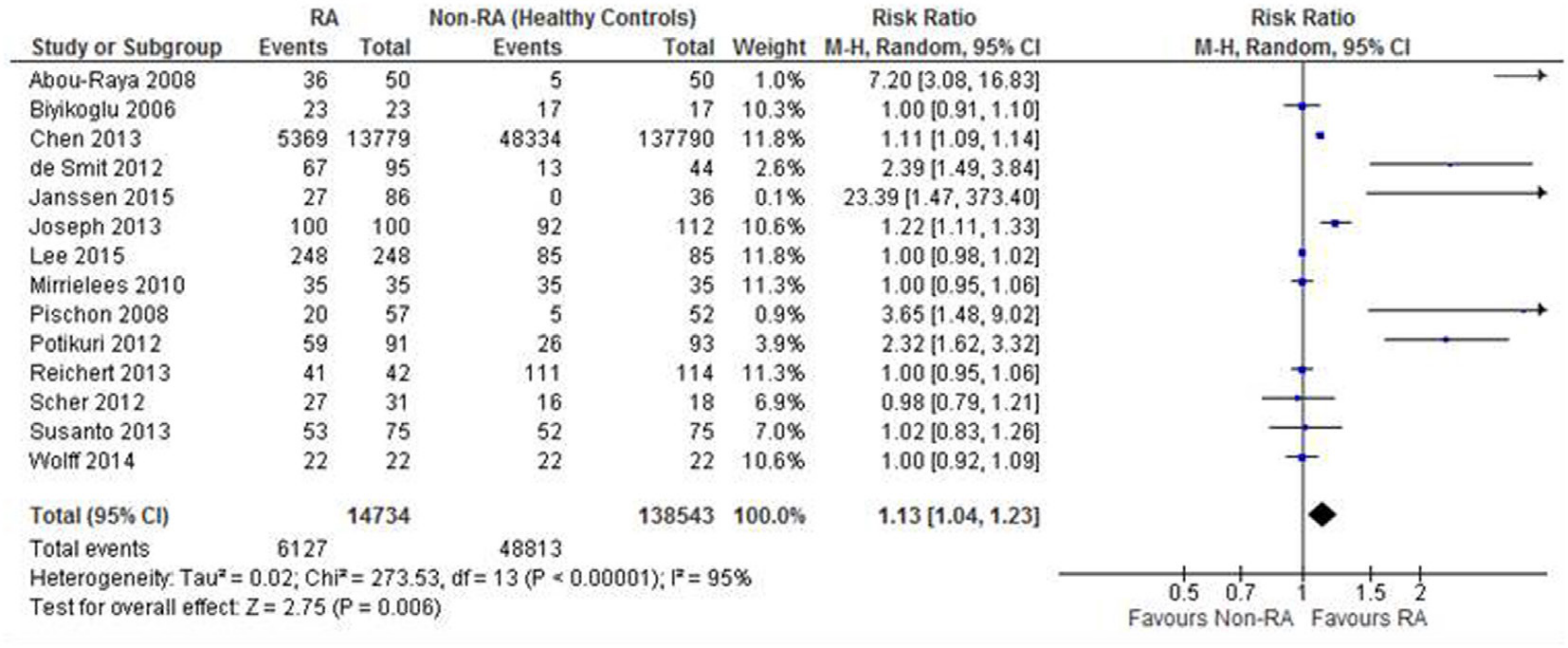
**Forest-plot representing risk ratio of periodontitis between patients with RA and healthy control comparisons**.

The assessment of episodes of periodontitis was assessed in 14 studies. There was a statistically significantly greater risk of periodontitis for people with RA compared to health comparable cohorts. Those with RA had a 13% greater risk of periodontitis compared to the non-RA cohort, ranging from 4 to 23% (RR: 1.13; 95% CI: 1.04, 1.23; *p* = 0.006; *N* = 153,277; Figure 2).

The 17 studies were included in the whole RA analysis but not all assessed periodontitis risk (as a whole), with 7 presenting data on specific features of periodontitis, including probing depth, plaque index, BOP, etc., which were reported in Table 3. This accounts for the difference in Figure 2 and the whole dataset that is presented in the characteristics of included studies. On secondary analysis, there was no statistically significant difference in the risk of the probing depth >5 mm (RR: 4.93; 95% CI: 0.84, 28.95) or plaque index as assessed through the frequency of participants >0.6 (RR: 1.23; 95% CI: 0.81, 1.88) or plaque index value (MD: 2.27; 95% CI: −0.16, 4.70) between the RA cohort than non-RA cohort.

There was a significantly greater risk in the RA cohort of the frequency of any BOP in the RA cohort compared to the non-RA cohort (RR: 2.65; 95% CI: 1.00, 7.02; *p* = 0.05), gingivitis index (MD: 0.30; 95% CI: 0.20, 0.41), mean loss of teeth (MD: 2.46; 95% CI: 0.30, 4.63), and periodontal bone loss (RR: 2.05; 95% CI: 1.40, 2.98). There was also a significant difference between the RA and non-RA cohort for probing depth with the RA cohort demonstrating a 0.69-mm greater probing depth than the non-RA cohort (MD: 0.69; 95% CI: 0.26, 1.12).

Although there was no statistically significant difference between the cohorts for the assessment of the frequency of CAL (RR: 3.63; 95% CI: 0.40; 32.88), the RA cohort presented with greater CAL when assessed as an absolute value (MD: 0.99; 95% CI: 0.38, 1.61).

### Meta-Analysis: Risk Ratio of Periodontitis in RA versus OA Participants

**Figure 3:**
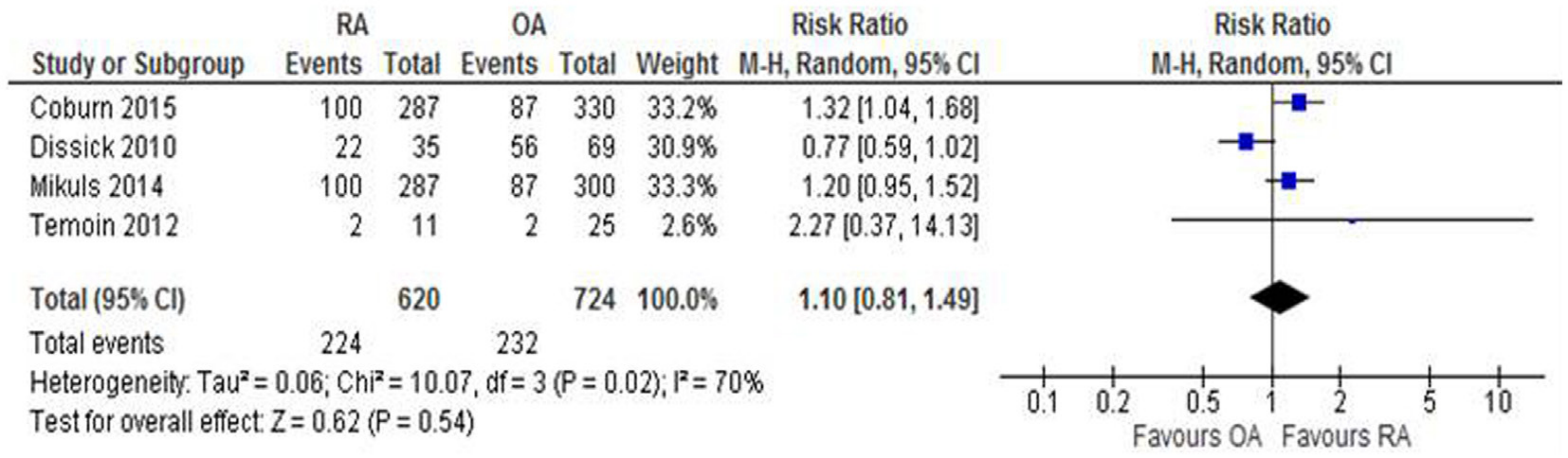
**Forest-plot representing risk ratio of periodontitis between patients with RA and OA participants**.

There was no statistically significant difference in risk of periodontitis between people with RA compared to those with OA (RR: 1.10; 95% CI: 0.81, 1.49; *p* = 0.54; *N* = 1344; Figure 3).

On secondary analysis, there was no statistically significant difference in the risk of the probing depth >5 mm in the RA cohort compared to the OA cohort (RR: 1.11; 95% CI: 0.95, 1.31; *p* = 0.19), the frequency of CAL (RR: 1.04; 95% CI: 0.74, 1.48; *p* = 0.81), or in the mean loss of teeth between those with RA compared to OA (MD: 0.10; 95% CI: −0.62, 0.42). There was, however, a significant difference in the risk of BOP, where the OA cohort presented with an 8% greater risk of exhibiting this feature of periodontitis compared to the RA cohort (RR: 0.92; 95% CI: 0.88, 0.97; *p* = 0.002).

## Discussion

We performed a meta-analysis to investigate the relationship between periodontitis and RA, finding that there was a significantly greater risk of periodontitis in people with RA when compared to healthy (non-RA) controls (RR 1.13, 95% CI: 1.04, 1.23; *p* = 0.006). This finding was not demonstrated when comparing the risk of periodontitis in RA and OA controls.

There was no significant difference in probing depths >5 mm in RA compared to healthy controls; however, there was a 0.69-mm greater probing depth in RA. Although the frequency of CAL did not significantly differ between RA and controls; however, the absolute value of CAL demonstrated a MD of 0.99 (95% CI: 0.38, 1.61). There was an increased risk of BOP in RA compared to healthy controls (2.65, 95% CI: 1.00, 7.02; *p* = 0.05) but, interestingly, a reduced risk compared to OA patients (0.92, 95% CI: 0.88, 0.97; *p* = 0.002).

In terms of oral hygiene parameters, gingival index was significantly higher in RA compared to healthy controls; however, this finding was not replicated in plaque index, another parameter of oral hygiene. There was a significantly higher mean loss of teeth in RA compared to controls (MD: 2.46, 95% CI: 0.30, 4.63).

Our primary finding that there is an increased risk of periodontitis in patients with RA compared to healthy controls is in consort with the findings of recent narrative reviews, but our study is the first meta-analysis of its kind interrogating this question. In 2015, Araújo and colleagues published a critical appraisal of studies investigating the relationship between RA and periodontitis (47). Papers published since 2012 were selected including eight epidemiological studies, four periodontal intervention studies, and five investigating the role of inflammatory mediators in both diseases. They found that 21 studies demonstrated an association though statistical analysis and 3 studies demonstrated an association through descriptive analysis between RA and periodontitis (47). We found an association between RA and periodontitis in our meta-analysis of papers published until October 2015 across an international dataset.

With respect to underlying pathophysiology, CP and RA share many pathological features. These include a number of factors, including oxygen metabolism (41), other shared mechanisms, including active and quiescent inflammatory phases, with the release of several mediators that are common to both conditions, including interleukin 1-beta and prostaglandin E2 (48–50). Similarly, collagenase is a metalloproteinase that specifically degrades collagen. It is not only detected at elevated levels in RA synovial fluid and in the circulation in subjects with RA but also found in gingival crevicular fluid (GCF) and gingival tissue (48). Collagenase activity is higher in GCF from subjects with periodontitis than healthy controls (49). Kobayashi et al. (50) reported that the disease activity of RA correlates with serum levels of IL-6, TNF alpha, and CRP, and the higher cytokine levels may influence BOP depth in RA in patients with moderate to high disease activity. Citrullination of auto-antigens is one of the hallmarks of RA and antibodies to cyclic citrullinated peptides are associated with more aggressive and erosive rheumatoid disease. The main human enzyme causing citrullination is peptidylarginine deiminase (PAD). The only known bacterial enzyme causing citrullination is PPAD, produced by *P. gingivalis*. *P gingivalis* forms part of the red complex of organisms implicated in periodontitis. Several studies worldwide have been conducted to investigate the presence of anti-*P. gingivalis* antibodies. Okada et al. (51) showed significantly higher levels of anti-Pg and anti-CCP antibodies than controls (*p* = 0.04 and *p* < 0.0001). In their study, the investigators also showed a significant association of anti-Pg responses with RA, after adjustment for age, sex, and smoking (*p* = 0.005 and *p* = 0.02), suggesting that serum levels of anti-Pg antibodies are associated with RA and might affect serum levels of RF and periodontal condition in patients with RA.

### Study Limitations

The analysis of periodontitis is complicated by differences in the definition of the disease, with some studies defining “mild/moderate/severe” periodontitis (31, 33, 35, 44, 52), others using clinical parameters of periodontitis, including probing depth (10, 29, 38, 40, 53, 54) and CAL (55). In none of the papers, a division of periodontitis into the aggressive and chronic phenotypes of the disease a fact that may be relevant as RA is more classically related to CP was found. Therefore, we recognize that our study has limitations, thus the results should be interpreted with caution.

Heterogeneity of disease is also an issue when considering the RA populations studied with marked variation in the range of disease duration from a mean of 3.4 months (37) to 16.3 years (34). The temporal relationship between RA and periodontitis is yet to be established and one of the difficulties in comparing the outcomes of these two studies would be the markedly different disease populations. The difference in disease activity was most commonly assessed by DAS28 score and varied from a mean score of 2.2 (31) to 7.21 (21), again, representing phenotypically distinct disease that may affect the relationship with periodontal inflammation. However, in terms of meta-analysis, it is useful to have the whole range of the disease spectrum represented.

Treatment regimens were often not stated; however, those that were did vary in terms of the use of DMARDs varying from 0% in the new-onset arthritis patients in Scher et al.’s study (37) and 79% of patients taking at least one DMARD in the study of Janssen and colleagues (31). There is also variation with regard to the type of DMARD used with 74% of participants taking hydroxychloroquine (56), 71% on methotrexate (31) and over half on biological therapy (32). These variations can be accounted for by geographical location and the variation in treatment vogue over the time span of the papers sampled for the meta-analysis. Variation in therapeutics may be relevant as it is possible that RA DMARD therapy could also attenuate periodontitis and thus a seeming reduction in association.

Smoking is a confounder for periodontitis (57) and RA (58) possibly due to the role of cigarette smoke in citrullination and the production of reactive oxygen species. As such it is perhaps limiting, in terms of adjustment for confounding, that seven of the studies selected did not comment on the current smoking status of participants.

The studies included in our analysis were not investigating cause and effect but rather establishing the point prevalence of periodontitis and RA. Further longitudinal trials are required to establish the temporal nature of this association.

Any systematic review investigating this disease association over a long time will be limited by geographical demography, changes in definition of disease, disease severity, and treatment regimens as they are modified over the years, which could lead to complications with the comparison of data from heterogeneous populations. However, within the context of a systematic review, this will simply broaden the relevance of our findings as the association appears to withstand changes in the above variables.

## Conclusion

We present a systematic review and meta-analysis of the relationship between periodontitis and RA, which demonstrates a significant association between RA and periodontitis. Further studies are required in future to elucidate the mechanism of this association.

## Author Contributions

NF, TS and NS conceived, analysed and drafted the manuscript. AK conducted literature searches and reviewed the manuscript. All authors approved the manuscript for publication.

## Conflict of Interest Statement

The authors declare that the research was conducted in the absence of any commercial or financial relationships that could be construed as a potential conflict of interest.
